# A Bidirectional Quasi-Endfire Patch Antenna with Low Elevation Angle

**DOI:** 10.3390/mi15060777

**Published:** 2024-06-12

**Authors:** Ziling Zhou, Jin Shi, Gu Liu, Kai Xu, Ruirui Jiang

**Affiliations:** 1 School of Information Science and Technology, Nantong University, Nantong 226019, China; zzlhj23731@outlook.com (Z.Z.); jinshi0601@hotmail.com (J.S.); lg354867769@outlook.com (G.L.); xukaihopeness@hotmail.com (K.X.); 2 Research Center for Intelligent Information Technology, Nantong University, Nantong 226019, China

**Keywords:** bidirectional radiation, low elevation angle, quasi-endfire, patch antenna

## Abstract

A bidirectional quasi-endfire patch antenna with a low elevation angle has promising applications for wireless communication systems that are vehicle-based, airborne, and shipborne. In this paper, the shortened patch resonators and open patch resonator are integrated to form a bidirectional quasi-endfire patch antenna with low elevation angle. The open patch resonator operates with a TM_20_ mode to realize bidirectional radiation. The two shortened patch resonators operate with a TM_01_ mode coupled with a TM_20_ mode to control the phase difference between them at a suitable angle, so that the shortened patch resonators act as directors to tilt the dual beams toward the endfire direction and achieve low elevation angle. Compared with reported patch antennas with dual beams, the proposed antenna has the lowest elevation angle and a compact structure. For demonstration purposes, an antenna prototype operating at 3.5 GHz is fabricated and measured, exhibiting a low elevation angle of ±28°, a −10 dB impedance matching bandwidth from 3.44 GHz to 3.61 GHz, and a size of 1.36 λ_0_ × 0.57 λ_0_ with a profile of 0.036 λ_0_. A prototype with two pair of shortened patch directors further reduces the elevation angle to ±19° with the size of 2.3 λ_0_ × 0.57 λ_0_.

## 1. Introduction

Antennas with dual beams and a low elevation angle can realize bidirectional quasi-endfire radiation, which has promising prospects for wireless communication systems that are vehicle-based, airborne, and shipborne. Meanwhile, such antennas usually have many small substructures that are far smaller than the operating wavelength, such substructures deriving from the field of engineering technology combined with micromanufacturing. The reported bidirectional endfire antennas [1,2,3,4,5,6] have no complete ground and can achieve bidirectional endfire radiation through the back-to-back configuration, but the patterns will be greatly disturbed by the metal plane in the systems. Therefore, research on bidirectional antennas with a low elevation angle and complete ground has important scientific significance and engineering value.

A patch antenna with dual beams and complete ground can achieve bidirectional quasi-endfire radiation. In [7], the influence of the substrate permittivity on the elevation angle of the dual beams was studied, and an elevation angle of 58° was found to be achievable. By introducing middle-layer strips under the radiating patch [8,9], the minimum elevation angle has been reduced to ±50°, but with a complex structure. Patches loaded with slots [10,11,12,13] have been shown to be able to avoid complex structures and attain a minimum elevation angle of 49° [12]. Bowtie-shaped patches loaded with metallic material via a wall along one edge [14] can achieve an elevation angle of ±45°. In [15], a quasi-radiator loaded with a fork slot and a shorting via was proposed, the beam tilting was realized through the overlap of two orthogonal fields, and the minimum elevation angle attained was 42°. A grounded patch antenna with three parasitic patches on the middle layer [16] was shown to attain a reduced elevation angle of ±40°. These designs have reduced the elevation angle to a certain degree, but the angle should be further reduced.

There are also some research studies on reducing the elevation angle in unidirectional quasi-endfire patch antennas. In [17], the partially reflecting surface was placed upon the cylindrical dielectric resonator to change the phase gradient and reduce the elevation angle to 55°, but with a high profile. A metasurface structure was also proposed in [18] to realize tilted beam radiation, with an elevation angle, again, of 55°. Most of the available research has reduced the elevation angle by combining reflector, director, and driver patches. Generally, the size of a reflector patch is larger than that of the driver patch, while the director patch is smaller than the driver patch [19,20,21,22,23]. In [24], the size of the reflector patch and director patch compared with the driver patch was exactly opposite, because the patches operated with the higher order TM_20_ mode. By loading slots on the reflector patch and pins on the director patches [25], unidirectional quasi-endfire radiation can be also achieved. There is also research on quasi-endfire patch antennas with an electromagnetic band gap structure as a reflector and a quarter-wavelength patch antenna with one edge shorted as a director [26]. In summary, the lowest elevation angle that these antennas can attain is 30°, but only with unidirectional radiation. Recently, the Butler matrix and a reconfigurable frequency selective surface [27] were used to reduce the elevation angle. The minimum elevation angle attainable was 18°. However, the structure was complex and its size large. In [28], a metasurface structure was designed using patch arrays with different sizes on a metallic surface, which can achieve a wideband and quasi-endfire radiation but with unidirectional radiation and a large size. A printed Yagi antenna employing elliptically shaped coupled directive elements can achieve wideband quasi-endfire radiation and attain a smaller size, but, again, with unidirectional radiation [29]. It is necessary to research a patch antenna that not only achieves a low elevation angle, but that can also achieve bidirectional radiation.

In this paper, a bidirectional quasi-endfire patch antenna with a low elevation angle is proposed. The operating modes and phase distribution are studied to give the working principle. Then, the key parameter analysis and a case study with two pairs of shortened patch resonators are presented, achieving the lowest elevation angle of ±19°. Finally, two prototypes of the proposed antennas are fabricated and measured.

## 2. Antenna Design

### 2.1. Antenna Configuration

**Figure 1:**
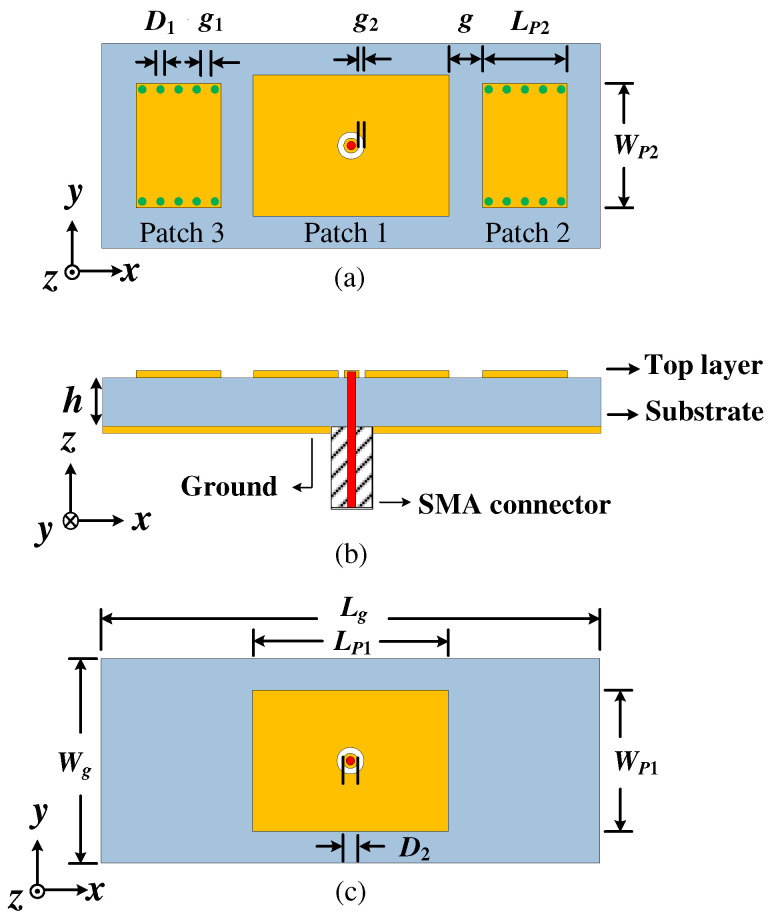
The configurations of the proposed Ant. 1. (**a**) Top view, (**b**) cross-section view, (**c**) top view of the single patch antenna.

Figure 1 gives the configurations of the proposed Ant. 1 and single patch antenna. Figure 1a is the top view of the proposed Ant. 1, which mainly consisted of three radiation patches, including an open rectangular patch located in the middle of the grounded dielectric substrate (denoted as Patch 1) and two shortened rectangular patches symmetrically placed on both sides of Patch 1 (denoted as Patch 2 and Patch 3). Patch 1 was loaded with an annular groove in the center and connected to the feeding probe at the center point, which was designed to operate at TM_20_ mode to generate bidirectional radiation. Therefore, the length of Patch 1 (*L_P_*_1_) should be *λ* [7], where *λ* is the wavelength. Patch 2 and Patch 3 operated at TM_01_ mode and were set as directors to pull the dual beams toward the endfire direction. The length of Patch 2/Patch 3 (*L_P_*_2_) was *λ*/2 [30]. A row of metallic vias were loaded on the upper and lower edges of Patch 2 and Patch 3 to suppress the radiation generated by the two shortened edges in the x-direction and ensure the stability of bidirectional quasi-endfire radiation within the operation frequency band. Patch 1 and the grounded dielectric substrate formed the open patch resonator. Patch 2/Patch 3 and the grounded dielectric substrate formed the bilateral shortened patch resonator. The cross-section view of the proposed Ant. 1 is shown in Figure 1b. The signal was fed into Patch 1 through the SMA connector, then coupled with Patch 2 and Patch 3 to achieve bidirectional quasi-endfire radiation. The dielectric substrate was RO4003C with a dielectric constant (*ε_r_*) of 3.38, a loss tangent (tan*δ*) of 0.0027, and a thickness of 3.148 mm. For comparation, a single patch antenna is given, which has the same dimensions as the proposed Ant. 1, except for the absence of Patch 2 and Patch 3. The top view of the single patch antenna is shown in Figure 1c. The professional simulation software CST 2017 was used to study the radiation performance of the proposed antennas in this work.

### 2.2. Antenna Operating Mechanism

**Figure 2:**
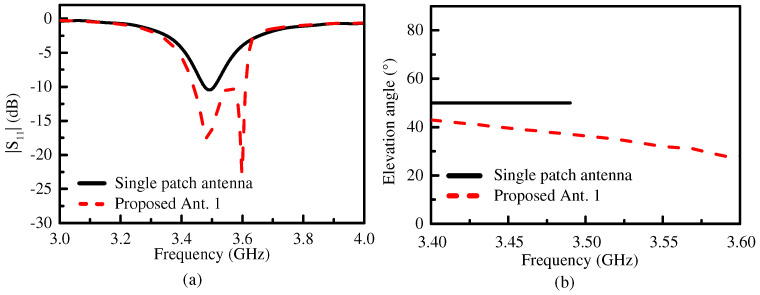
The simulated (**a**) |S_11_| and (**b**) the elevation angles of the proposed Ant. 1 and the single patch antenna within their respective operating bandwidth.

Figure 2a compares the simulated |S_11_| of the proposed Ant. 1 and the single patch antenna. The operating bandwidth (with |S_11_| < −10 dB) of the proposed Ant. 1 was about 4.8% (from 3.44 GHz to 3.61 GHz), which is significantly extended compared with the operating bandwidth of the single patch antenna (from 3.47 GHz to 3.51 GHz). From the simulation results, we can see that there were two reflection zeros of the proposed Ant. 1 at the frequencies of 3.49 GHz and 3.59 GHz, while the single patch antenna had only one reflection zero at 3.48 GHz. Figure 2b shows that the elevation angle of the proposed Ant. 1 ranged from ±40° to ±28° within the operating bandwidth, which is clearly lower than the 50° of the single patch antenna. The elevation angle of the single patch antenna remained unchanged within the operating bandwidth, and the elevation angle of the proposed Ant. 1 decreased with the frequency increase.

**Figure 3:**
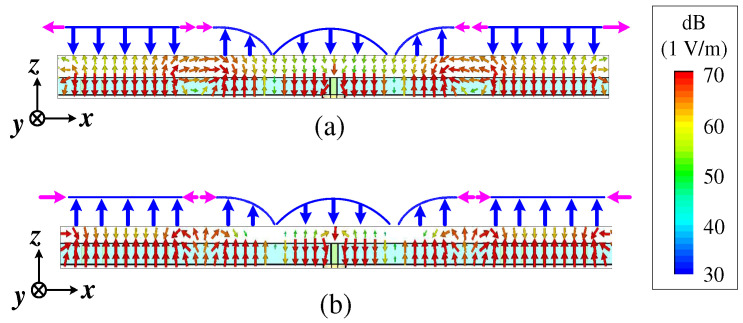
The simulated vector’s electric field distribution of the proposed Ant. 1. (**a**) At the first reflection zero (at 3.49 GHz), (**b**) at the second reflection zero (at 3.59 GHz).

Figure 3 shows the simulated vector’s electric field distribution of the proposed Ant. 1. It is clear that the internal electric field distribution of Patch 1 was on TM_20_ mode: the electric field was a full-wave distribution in the x direction, while, in the y direction, the direction of the electric field was the same and the amplitude was equal. The internal electric field distribution of Patch 2 and Patch 3 was on TM_01_ mode, the direction of the electric field was the same and the amplitude was equal in the x direction, while the electric field exhibited a half-wave distribution in the y direction. The horizontal components of the electric field of the TM_20_ mode on both sides of Patch 1 had equal amplitudes and opposite directions, so the far-field radiation pattern was offset from the broadside but superimposed along the endfire direction, which can generate the symmetrical dual-beam radiation. The internal electric field of the TM_01_ mode was of equal amplitude and in the same direction, the horizontal electric field components on the left and right sides of Patch 2 and Patch 3 had equal amplitudes and a phase difference of 180°, which can maintain the bidirectional radiation of the proposed Ant. 1. Meanwhile, two reflection zeros were formed due to the coupling of the TM_20_ mode with the TM_01_ mode, a phenomenon which is consistent with the simulation results in Figure 2a. At the first reflection zero (Figure 3a), the electric field of the two modes was almost reversed (blue arrow), and the electric field amplitude of the TM_01_ mode was smaller than that of the TM_20_ mode. The direction of the horizontal components of the electric field near two adjacent resonators was similar (pink arrow). At the second reflection zero (Figure 3b), the electric field of the two modes was similar, and the electric field amplitude of the TM_01_ mode was equal to that of the TM_20_ mode. The direction of the horizontal components of the electric field near two adjacent resonators was reversed. The change of coupling with the frequency resulted in the change of the elevation angle, confirming the results in Figure 2b.

**Figure 4:**
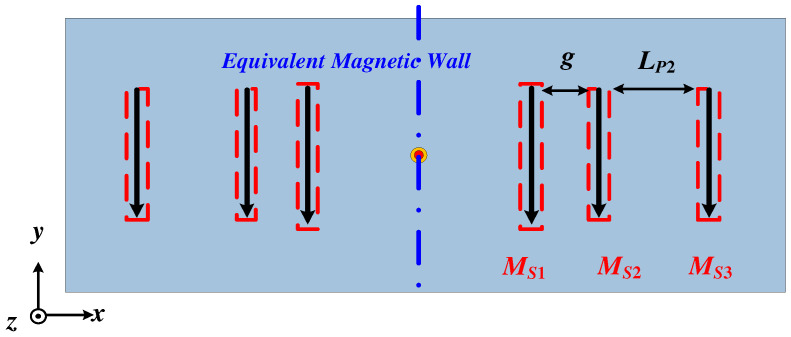
The equivalent magnetic currents of the proposed Ant. 1.

According to the antenna radiation theory [30], the radiation pattern of a patch antenna can be equivalent to the radiation superposition of the magnetic currents in two slots located along two radiation edges of the patch. Thus, the proposed Ant. 1 can be equivalent to six magnetic current sources, as shown in Figure 4. Meanwhile, the whole antenna is center-symmetric, and the central plane is equivalent to a magnetic wall, so that the far-field electric field of the right half part can be calculated using a three-element slots array, expressed as *M_S_*_1_, *M_S_*_2_, and *M_S_*_3_. The distances separating the three adjacent slots are *g* and *L_P_*_2_. Hence, the far-field electric field radiated by *M_S_*_1_, *M_S_*_2_, and *M_S_*_3_ can be expressed as:

(1) $$F_{E}\left(\varphi \right)=E_{1\phi }+E_{2\phi }+E_{3\phi }=E_{1\phi }\left(1+\frac{M_{S2}}{M_{S1}}e^{jkg\sin\theta \sin\varphi }+\frac{M_{S3}}{M_{S1}}e^{jk\left(g+L_{P2}\right)\sin\theta \sin\varphi }\right)$$

where *F*_E_(*φ*) is the amplitude of the far-field electric field, *E*_1ϕ_, *E*_2ϕ_, *E*_3ϕ_ are the far-field electric fields induced by *M_S_*_1_, *M_S_*_2_, and *M_S_*_3_, respectively, and *φ* is the beam tilt angle.

**Figure 5:**
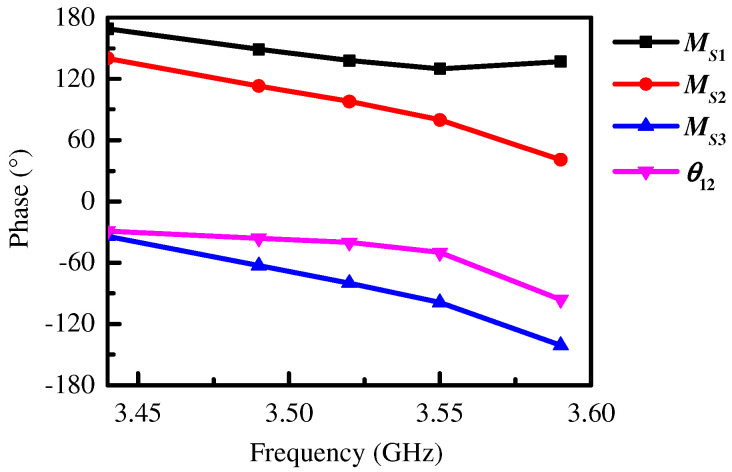
The simulated phases of *M_S_*_1_, *M_S_*_2_, and *M_S_*_3_, and phase difference *θ*_12_ between *M_S_*_2_ and *M_S_*_1_.

According to the analysis of the working modes of the proposed antenna, we know that Patch 2 operated on TM_01_ mode, so the magnetic currents *M_S_*_2_ and *M_S_*_3_ maintained the same amplitude and 180° phase difference. Figure 5 also gives the simulated phases of *M_S_*_1_, *M_S_*_2_, and *M_S_*_3_ within the operating bandwidth, from which we can see that the phase difference between *M_S_*_2_ and *M_S_*_3_ was close to 180°, that is:

(2) $$M_{S3}=M_{S2}e^{j\pi }$$

Assuming that the ratio of the amplitudes between *M_S_*_2_ and *M_S_*_1_ is *a*_1_, and the phase difference between them is *θ*_12_:

(3) $$M_{S2}=a_{1}M_{S1}e^{j\theta_{12}}$$

Thus, the electric field strength in the far field of the three equivalent magnetic currents can be obtained as:

(4) $$F_{E}\left(\varphi \right)=E_{1\phi }\left(1+a_{1}e^{j\left(kg\sin\theta \sin\varphi +\theta_{12}\right)}+a_{1}e^{j\left[k\left(g+L_{P2}\right)\sin\theta \sin\varphi +\theta_{12}+\pi \right]}\right)$$

In the *E*-plane (*θ* = 90°), the far-field electric field of the right half antenna is:

(5) $$F_{E}\left(\varphi \right)=E_{1\phi }\left(1+a_{1}e^{j\left(kg\sin\varphi +\theta_{12}\right)}+a_{1}e^{j\left[k\left(g+L_{P2}\right)\sin\varphi +\theta_{12}+\pi \right]}\right)$$

From (5), we can conclude that the beam tilt angle (*φ*) is a function of *g*, *L_P_*_2_, *a*_1_, and *θ*_12_. Among them, *a*_1_ and *θ*_12_ are the key factors affecting radiation pattern, while the value of *g* and *L_P_*_2_ will influence *a*_1_ and *θ*_12_, but *a*_1_ has only a small effect on the beam angle. In this work, we mainly focused on the effect of *θ*_12_ on the elevation angle. When −180° < *θ*_12_ < 0°, the other parameters were fixed, with Patch 2 acting as a director. When 0° < *θ*_12_ < 180° and the other parameters were fixed, Patch 2 acted as a reflector. As the value of *θ*_12_ decreased, the radiation beam moved from a broadside direction to an endfire direction, meaning that the elevation angle was reduced.

**Figure 6:**
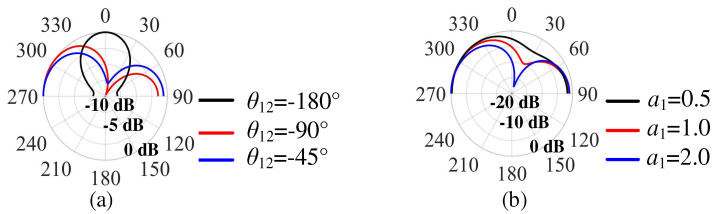
The calculated *E*-plane radiation patterns (**a**) with different *θ*_12_, (**b**) with different *a*_1_.

Figure 6 gives the calculated *E*-plane radiation patterns with different *θ*_12_ and *a*_1_ according to (5). Figure 6a illustrates the case of −180° < *θ*_12_ < 0°, with the radiation beam moving from a broadside direction to an endfire direction, indicating that Patch 2 acted as director. The elevation angle decreased with increasing *θ*_12_. The elevation angle remained almost unchanged when *a*_1_ increased from 0.5 to 2, as shown in Figure 6b.

**Figure 7:**
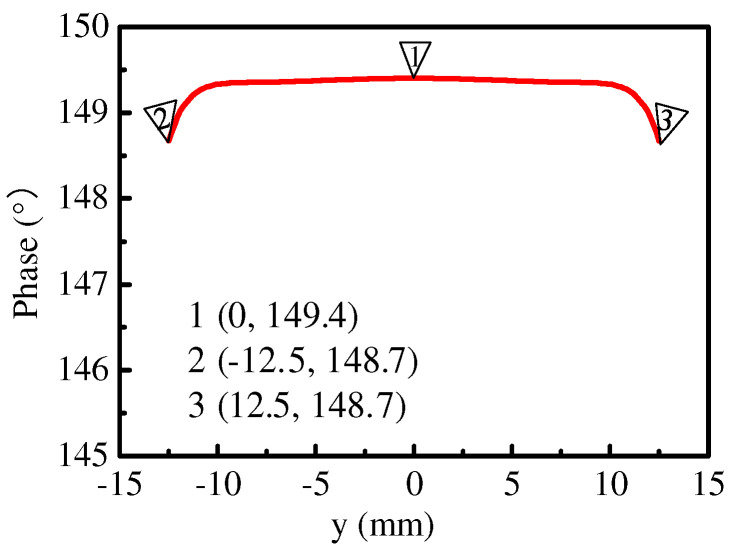
The phase distribution along the slot width (*M_S_*_1_) at 3.49 GHz.

The simulated phase difference *θ*_12_ between *M_S_*_2_ and *M_S_*_1_ is also given in Figure 5. Here, the phase of the equivalent magnetic current was calculated by the midpoint of the corresponding slot width in the simulation because the phase distribution along the slot width was uniform, as shown in Figure 7. We can see that the value of *θ*_12_ was in the range of −180° < *θ*_12_ < 0°, indicating that Patch 2 played the role of director. Similarly, we can conclude that Patch 3 also acted as a director, causing the dual beams of the proposed Ant. 1 to be tilted toward the endfire direction at the same time and the elevation angle of the proposed Ant. 1 to be reduced. Furthermore, the elevation angle was reduced in the operating bandwidth because the value of *θ*_12_ decreased with increasing frequency. The simulation results in Figure 2b are consistent with the above analysis.

### 2.3. Parametric Analysis on L_P2_, W_P2_, and g

**Figure 8:**
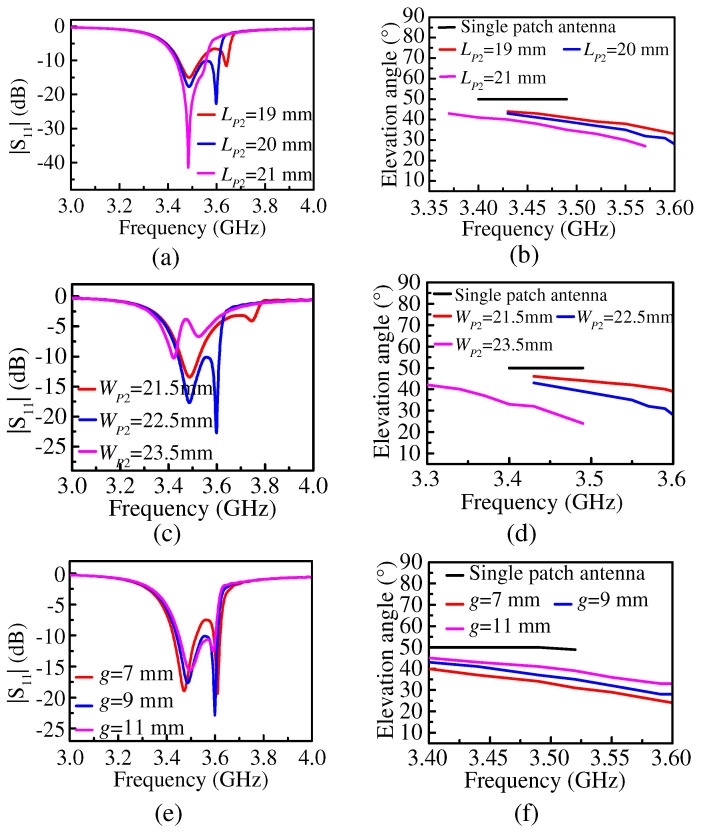
(**a**) The simulated |S_11_| and (**b**) the elevation angle of the proposed Ant. 1 under different *L_P_*_2_. (**c**) The simulated |S_11_| and (**d**) the elevation angle of the proposed Ant. 1 under different *W_P_*_2_. (**e**) The simulated |S_11_| and (**f**) the elevation angle of the proposed Ant. 1 under different *g*.

Subsequently, the key parameters that affect |S_11_| and the elevation angle of the proposed Ant. 1 were analyzed, such parameters mainly including the length of the shortened patch *L_P_*_2_, the width of the shortened patch *W_P_*_2_, and the distance between the shortened patch and the open patch *g*, as shown in Figure 8.

From Figure 8a, we can see that the second reflection zero decreased with an increase in *L_P_*_2_. This is because the value of *L_P_*_2_ affected the resonant frequency of Patch 2. As the value of *L_P_*_2_ increased, the resonant frequency of Patch 2 decreased. When the value of *L_P_*_2_ varied from 19 mm to 21mm while keeping the other parameters fixed, the minimum elevation angle decreased from 32° to 27°, as shown in Figure 8b, and it gradually tilted away from the broadside direction toward the endfire direction. The reason is that the coupling of Patch 2 was enhanced with the increase in *L_P_*_2_. However, when the value of *L_P_*_2_ was too large, the second reflection zero moved closer to the first reflection zero, and the operating bandwidth of the proposed antenna became narrower. When the value of *L_P_*_2_ was too small, the distance between the two reflection zeros was large, with impedance mismatching somewhere in the operating bandwidth and a narrow operating bandwidth. Therefore, it is necessary to choose an appropriate value of *L_P_*_2_ to ensure a wide operating bandwidth while reducing the elevation angle. In this work, the value of *L_P_*_2_ was selected to be 20 mm based on the simulation results.

From Figure 8c, we can see that the second reflection zero decreased with an increase in *W_P_*_2_, because the value of *W_P_*_2_ affected the resonance frequency of Patch 2. As *W_P_*_2_ increased, the resonant frequency of Patch 2 decreased and the elevation angle decreased with the increase in *W_P_*_2_. As the value of *W_P_*_2_ increased from 21.5 mm to 23.5 mm while keeping the other parameters fixed, the minimum elevation angle decreased from 39° to 24°, as shown in Figure 8d. This is because the coupling of Patch 2 was enhanced with an increase in *W_P_*_2_. However, if the value of *W_P_*_2_ was too small, the distance between the two reflection zeros would be too large, splitting the operating bandwidth and reducing the matching bandwidth. If the value of *W_P_*_2_ was too large, the reflection performance of the proposed antenna would deteriorate sharply. Based on the aforementioned analysis, there was a tradeoff between the low elevation angle and a wide matching bandwidth. From the simulation results, we can see that a value of 22.5 mm was suitable for *W_P_*_2_.

The elevation angle decreased with a decrease in *g*, as shown in Figure 8f. As the value of *g* decreased from 11 mm to 7 mm while keeping the other parameters fixed, the minimum elevation angle decreased from 33° to 25°. This is because the coupling between Patch 1 and Patch 2 was stronger with a decrease in *g*. Meanwhile, the frequency spacing between the two resonant points increased with a decrease in *g*, resulting in deteriorated impedance matching, as shown in Figure 8e. Therefore, the value of *g* should be selected after considering the low elevation angle and good impedance matching bandwidth. After comprehensive consideration, the value of *g* was selected to be 9 mm.

### 2.4. Research on the Bidirectional Quasi-Endfire Patch Antenna with Multiple Shortened Patches

**Figure 9:**
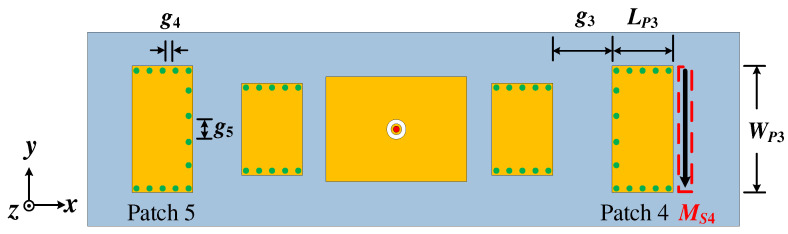
The configuration of the proposed Ant. 2.

Through the above research, the proposed Ant. 1 loaded with a pair of shortened patches on both sides, equivalent to two magnetic current sources on both sides, was shown to reduce the elevation angle. Based on this, we researched the patch antenna loading with three equivalent magnetic current sources on both sides to further reduce the elevation angle. A patch antenna (the proposed Ant. 2) loaded with an extra pair of shortened patches (Patch 4 and Patch 5) with three edges operating on TM_01_ mode was proposed, where Patch 4 or Patch 5 were equivalent to one magnetic current source. So, the calculation for the size of Patch 4/Patch 5 is like that of the half-mode SIW [31]. The configuration of the proposed Ant. 2 is given in Figure 9. Similarly, the patch resonator that can provide two or three magnetic currents can also be used as a director; however, this is not described in detail in this work.

**Figure 10:**
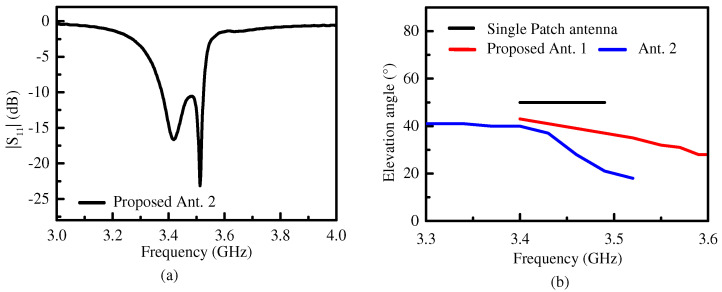
(**a**) The simulated |S_11_| of the proposed Ant. 2. (**b**) Elevation angles of the single-patch antenna, the proposed Ant. 1, and the proposed Ant. 2 in their respective operating bandwidths.

Figure 10a is the simulated |S_11_| of the proposed Ant. 2. There are two reflection zeros and the −10 dB impedance matching bandwidth ranged from 3.37 GHz to 3.52 GHz and corresponded to 4.3% in fraction. As shown in Figure 10b, the lowest elevation angle of 19° was lower than that of the proposed Ant. 1. The elevation angle gradually decreased from 40° to 19° as the frequency increased. This is because the coupling of *M_S_*_3_ with *M_S_*_4_ in relation to the proposed Ant. 2 increased with increasing frequency, so the elevation angle was further reduced, although the overall size was larger than that of the proposed Ant. 1.

## 3. Results and Discussion

**Figure 11:**
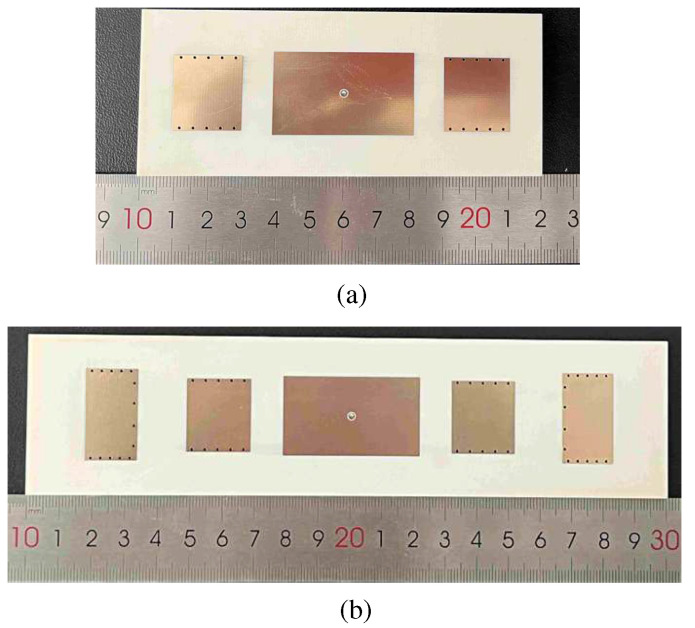
The prototype of (**a**) the proposed Ant. 1 and (**b**) the proposed Ant. 2.

In order to verify the radiation characteristics of the proposed bidirectional quasi-endfire patch antenna, based on the above analysis, two prototypes of the two proposed antennas were designed and measured, as shown in Figure 11. The detailed dimensions of the proposed Ant. 1 were: *L_g_* = 120, *W_g_* = 50, *L_P_*_1_ = 42, *W_P_*_1_ = 25, *L_P_*_2_ = 20, *W_P_*_2_ = 22.5, *D*_1_ = 0.8, *g* = 9, *g*_1_ = 3.2, *D*_2_ = 2.1, *g*_2_ = 0.3, and *h* = 3.148, where the unit is mm. The detailed dimensions of the proposed Ant. 2 were: *L_g_* = 200, *W_g_* = 50, *L_P_*_1_ = 43, *W_P_*_1_ = 25, *L_P_*_2_ = 20, *W_P_*_2_ = 23, *L_P_*_3_ = 16, *W_P_*_3_ = 28.5, *g* = 10, *D*_1_ = 0.8, *g*_1_ = 3.2, *D*_2_ = 2.1, *g*_2_ = 0.3, *g*_3_ = 15, *g*_4_ = 2.2, and *g*_5_ = 6.2, where the unit is mm.

**Figure 12:**
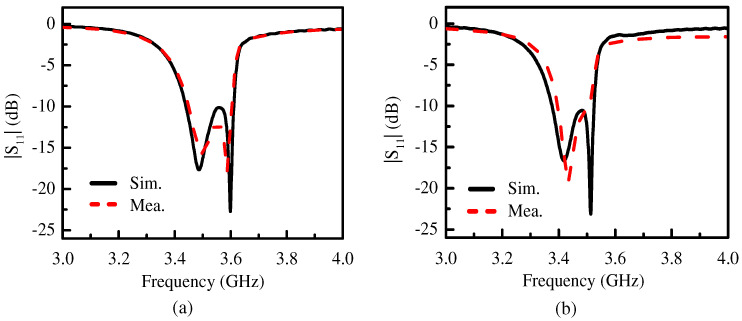
The simulated and measured |S_11_| of (**a**) the proposed Ant. 1 and (**b**) the proposed Ant. 2.

The S-parameter was measured using the Keysight N5230C vector network analyzer (Keysight Technologies, Santa Clara, CA, USA). The radiation patterns and the beam elevation angles were measured inside an anechoic chamber with a far-field antenna measurement system. Figure 12 shows the simulated and measured |S_11_| of two proposed antennas. The −10 dB impedance matching bandwidth of the proposed Ant. 1 was about 4.3% (3.45–3.6 GHz), as shown in Figure 12a, and the peak gain of the proposed Ant. 1 was about 5.2 dBi. The −10 dB impedance matching bandwidth of the proposed Ant. 2 was about 3.2% (3.4–3.51 GHz), as shown in Figure 12b, and the peak gain of the proposed Ant. 2 was 6.0 dBi. The small discrepancy between the simulated and measured results was due to the manufacturing tolerance and the measurement error.

**Figure 13:**
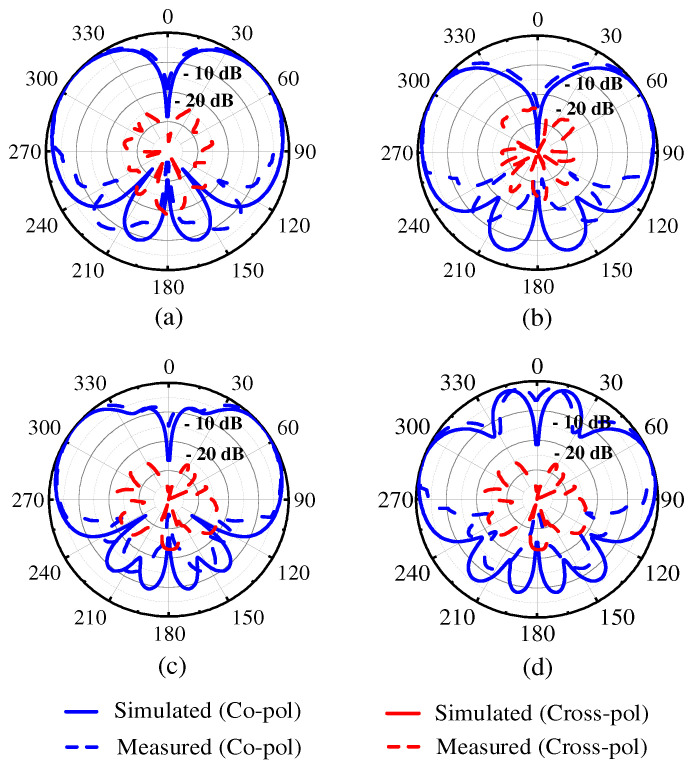
The simulated and measured radiation patterns on the *E*-plane of the two proposed antennas at two reflection zeros. (**a**) The proposed Ant. 1 at 3.49 GHz, (**b**) the proposed Ant. 1 at 3.59 GHz, (**c**) the proposed Ant. 2 at 3.42 GHz, and (**d**) the proposed Ant. 2 at 3.51 GHz.

Figure 13 illustrates the simulated and measured radiation patterns of two proposed antennas on the *E*-plane at two reflection zeros. For the proposed Ant. 1, the elevation angles at two reflection zeros were ±40° and ±28°, as shown in Figure 13a,b. For the proposed Ant. 2, the elevation angles at two reflection zeros were ±40° and ±19°, as shown in Figure 13c,d. The measured cross polarization levels were both below −20 dB.

**Figure 14:**
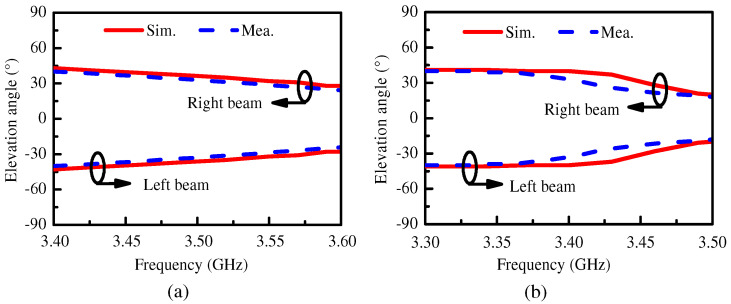
The simulated and measured elevation angles within the operating bandwidth of (**a**) the proposed Ant. 1 and (**b**) the proposed Ant. 2.

Figure 14 presents the simulated and measured elevation angles within the operating bandwidth for the two proposed antennas. From Figure 14, it can be seen that the elevation angle of the proposed Ant. 1 ranged from ±40° to ±28° in the operating bandwidth and the elevation angle of the proposed Ant. 2 ranged from ±40° to ±19° in the operating bandwidth. The elevation angle decreased with increasing frequency. The measured results are in agreement with the simulated outputs.

Table 1 provides the comparison between the proposed bidirectional quasi-endfire patch antennas and some reported designs. Compared with these designs, the proposed antennas attained the lowest elevation angle while retaining a compact structure. The level of manufacturing complexity for the antennas was divided into high (H), medium (M), and low (L), determined by considering the layers and the structure complexity of the designed antenna in the manufacturing process.

## 4. Conclusions

In this paper, a bidirectional quasi-endfire patch antenna with a low elevation angle is proposed. By controlling the mutual coupling between the TM_01_ mode and the TM_20_ mode, the phase distribution on the radiation edges met the requirement of bidirectional quasi-endfire radiation and attained a low elevation angle of ±28°. Furthermore, the lowest elevation angle of ±19° was obtained by using two pair of shortened patches to improve the control ability of the beam angle. Compared with the reported patch antennas with dual beams, the proposed design can attain the lowest elevation angle, which is suitable for wireless communication systems that are airborne, shipborne, and vehicle-based.

## Figures and Tables

**Table 1:** Performance summary of the proposed antennas and state-of-the art designs.

Ref.	*f*_0_^1^ (GHz)	Patch Dimensions (λ_0_ × λ_0_^2^)	FBW ^3^ (%)	Profile (λ_0_)	EA ^4^ (°)	The Lowest EA (°)	Dual Beams	MCL ^5^
[7]	5.46	0.77 × 0.44	13.2	0.1	±58~±61	±58	Y	M
[9]	5.4	0.8 × 0.46	19.4	0.043	±50	±50	Y	H
[12]	5.5	0.62 × 0.49	11.8	0.058	49~53	49	Y	L
[14]	5.9	0.67 × 0.67	3	0.03	±45	±45	Y	M
[16]	5.5	0.57 × 0.48	17.5	0.04	±40	±40	Y	H
[25]	5.1	1.5 × 1	11.5	0.025	30~40	30	N	L
[26]	4.8	2.1 × 1.12	3.3	0.04	30~40	30	N	M
Ant. 1	3.5	1.36 × 0.57	4.8	0.036	±28~±40	±28	Y	L
Ant. 2	3.45	2.3 × 0.57	4.3	0.036	±19~±40	±19	Y	L

^1^ *f*_0_: center frequency. ^2^ *λ*_0_: the wavelength in the free space at the center frequency. ^3^ FBW: 10-dB fractional bandwidth. ^4^ EA: elevation angle. ^5^ MCL: manufacturing complexity level.

## Data Availability

The data presented in this study are available upon request from the corresponding author.
